# Evaluation of the Molecular Docking of Potential Targets and the Time-Dependent Myocardial Effects of Omeprazole in Normotensive and Spontaneously Hypertensive Rats Subjected to Cardiac Ischemia and Reperfusion

**DOI:** 10.3390/ijms27135913

**Published:** 2026-06-30

**Authors:** Geraldo Teotônio de Aquino Filho, Alex Sandro Felisberto Oliveira, Erisvaldo Amarante de Araújo, Joyce Umbelino da Silva Yamamoto, Leiz Maria Costa Véras, Paulo Sérgio de Araujo Sousa, Jefferson Almeida Rocha, Adriano Caixeta, Mariana Chisté Ferreira, Isadora S. Rocco, Nelson Américo Hossne Junior, Solange Guizilini, Walter José Gomes, Afonso Caricati-Neto, Fernando Augusto Mardiros Herbella, Fernando Sabia Tallo, Célia Maria Camelo Silva, Rafael Guzella de Carvalho, Francisco Sandro Menezes-Rodrigues

**Affiliations:** 1 Postgraduate Program in Interdisciplinary Surgical Science, Universidade Federal de São Paulo (UNIFESP), São Paulo 04024-002, SP, Brazil; geraldo.aquino.filho@outlook.com (G.T.d.A.F.); alex.oliveira@unifesp.br (A.S.F.O.); fherbella@gmail.com (F.A.M.H.); fstallo1972@gmail.com (F.S.T.);; 2 Postgraduate Program in Cardiology, Universidade Federal de São Paulo (UNIFESP), São Paulo 04024-000, SP, Brazil; amarante.araujo@unifesp.br (E.A.d.A.); joyce.yamamoto@huhsp.org.br (J.U.d.S.Y.); adriano.caixeta@unifesp.br (A.C.); isadora.rocco@unifesp.br (I.S.R.); nelson.hossne@gmail.com (N.A.H.J.); wjgomes1012@gmail.com (W.J.G.); 3 Research Center on Biodiversity and Biotechnology, Universidade Federal do Delta do Parnaíba (UFDPar), Parnaíba 64202-020, PI, Brazil; leiz@ufpi.edu.br (L.M.C.V.); psergio.araujosousa@gmail.com (P.S.d.A.S.); jeffersonbiotec@gmail.com (J.A.R.); 4 Medicinal Chemistry and Biotechnology Research Group (QUIMEBIO), Universidade Federal do Maranhão (UFMA), São Bernardo 65550-000, MA, Brazil; 5 Department of Surgery, Discipline of Cardiovascular Surgery, Escola Paulista de Medicina, Universidade Federal de São Paulo (UNIFESP), São Paulo 04024-000, SP, Brazil; chistemariana@gmail.com; 6 Department of Pharmacology, Universidade Federal de São Paulo (UNIFESP), São Paulo 04023-062, SP, Brazil; caricatineto@gmail.com

**Keywords:** omeprazole, proton pump inhibitors, myocardial ischemia–reperfusion, hypertension, cardiotoxicity

## Abstract

Proton pump inhibitors (PPIs) are widely prescribed for acid-related disorders and are generally considered safe for short-term use. However, increasing experimental and clinical evidence suggests potential cardiovascular effects associated with chronic exposure, possibly related to endothelial dysfunction and impaired nitric oxide bioavailability. Therefore, we decided to investigate whether the cardiovascular effects of omeprazole are dependent on the timing of administration in a model of cardiac ischemia–reperfusion (CIR) in normotensive Wistar rats (NWR) and spontaneously hypertensive rats (SHR). Twelve- to sixteen-week-old male NWR and SHR were allocated into four groups: (1) SHAM: NWR and SHR were submitted to surgery with no ischemia; (2) (SS + CIR): NWR and SHR were treated with a 0.9% saline solution and submitted to CIR; and (3) (OME + ISQ): NWR and SHR were treated with 10 mg/kg i.v. omeprazole (OME) before cardiac ischemia and submitted to CIR or (4) after cardiac ischemia but before cardiac reperfusion (ISQ + OME). Electrocardiograms were monitored to assess ventricular arrhythmias (VA), atrioventricular block (AVB), and lethality (LET). Serum creatine kinase-MB (CK-MB) levels were quantified, and histopathological analyses were performed to evaluate the degree of myocardial injury in the different study groups. Administration of OME prior to cardiac ischemia increased the incidence of VA, AVB, LET, and serum CK-MB levels in both NWR and SHR. In contrast, administration before cardiac reperfusion did not exacerbate cardiac injury and was associated with the attenuation of electrophysiological instability. Histopathological findings corroborated the biochemical and functional outcomes. OME, when administered prior to cardiac ischemia, worsens both cardiac arrhythmias and myocardial injury; however, administration immediately prior to cardiac reperfusion does not increase cardiac arrhythmias and decreases myocardial injury in both NWR and SHR.

## 1. Introduction

Proton pump inhibitors (PPIs) are among the most widely prescribed medications worldwide due to their high efficacy in suppressing gastric acid secretion and their favorable short-term safety profile [1]. Since the introduction of omeprazole into clinical practice in the late 1980s, PPIs have become the cornerstone therapy for acid-related disorders, including gastroesophageal reflux disease, peptic ulcer disease, and eradication regimens for *Helicobacter pylori* infection [2]. Despite their gastrointestinal benefits, increasing concerns have emerged regarding potential extra-gastric effects associated with prolonged or inappropriate use.

Accumulating epidemiological evidence has suggested a possible association between chronic PPI use and adverse cardiovascular outcomes, including myocardial infarction and stroke [3,4]. Although these observational findings do not establish causality, they have stimulated investigation into the biological plausibility of cardiovascular effects mediated by PPIs. One of the most consistently proposed mechanisms involves endothelial dysfunction. Endothelial homeostasis depends critically on nitric oxide (NO) bioavailability, which regulates vascular tone, inhibits platelet aggregation, and modulates inflammatory signaling [5]. Experimental studies have demonstrated that PPIs can inhibit dimethylarginine dimethylaminohydrolase 1 (DDAH1), leading to the accumulation of asymmetric dimethylarginine (ADMA), an endogenous inhibitor of endothelial nitric oxide synthase (eNOS) [6]. Inhibition of the DDAH1/ADMA/eNOS pathway reduces NO bioavailability and promotes oxidative stress and vascular dysfunction, thereby increasing cardiovascular vulnerability [6].

In addition to endothelial impairment, emerging evidence suggests that PPIs may interfere with mitochondrial bioenergetics and redox homeostasis. Omeprazole has been shown to alter mitochondrial membrane potential and attenuate oxidative phosphorylation in non-gastric tissues, increasing susceptibility to oxidative stress and apoptotic signaling [7,8]. These mechanisms are particularly relevant in pathological settings characterized by abrupt metabolic stress, such as cardiac ischemia and reperfusion (CIR).

Myocardial CIR injury represents a complex pathophysiological process in which the restoration of coronary blood flow paradoxically contributes to additional tissue damage. Reperfusion is associated with the excessive generation of reactive oxygen species, calcium overload, endothelial dysfunction, inflammation, and the activation of cell death pathways [9,10]. The magnitude of myocardial injury during CIR is influenced by baseline endothelial function, mitochondrial integrity, and the activation of endogenous cardioprotective signaling pathways.

Hypertension is a major determinant of cardiovascular risk and is strongly associated with endothelial dysfunction and oxidative stress. Spontaneously hypertensive rats (SHR) constitute a well-established experimental model of essential hypertension, exhibiting impaired NO signaling, increased sympathetic activity, and enhanced susceptibility to ischemic injury [11,12]. Thus, the SHR provide a translationally relevant platform to investigate the pharmacological modulation of myocardial CIR under conditions of increased cardiovascular vulnerability.

Despite growing interest in the cardiovascular safety of PPIs, the impact of omeprazole on myocardial CIR injury remains incompletely understood. Importantly, the timing of drug administration relative to the ischemic insult has received limited attention. Pharmacological interventions may exert divergent effects depending on whether they interfere with pre-ischemic adaptive mechanisms or modulate oxidative and inflammatory cascades during reperfusion [13].

Therefore, the present study aimed to investigate the timing-dependent cardiovascular effects of omeprazole in normotensive and spontaneously hypertensive rats subjected to myocardial ischemia and reperfusion. By evaluating electrophysiological disturbances, biochemical markers of myocardial injury, and histopathological alterations, this study sought to determine whether omeprazole exacerbates or attenuates myocardial damage when administered before ischemia or immediately prior to reperfusion. Clarifying these context-dependent effects may provide important mechanistic and translational insights into the cardiovascular safety profile of proton pump inhibitors.

## 2. Results

### 2.1. Electrophysiological Ventricular Arrhythmias (VA) and Atrioventricular Block (AVB) and Letahlity Outcomes (LET) in Normotensive Wistar Rats (NWR)

**Figure 1:**
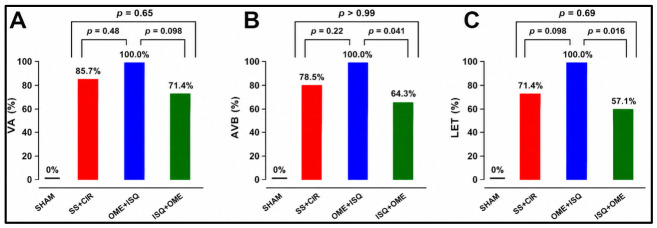
Incidence of (**A**) ventricular arrhythmias (VA), (**B**) atrioventricular block (AVB) and (**C**) lethality (LET) in normotensive Wistar rats (NWR) from SHAM, SS + CIR, OME + ISQ and ISQ + OME groups. Results are expressed as percentages obtained with 10–14 animals. Statistical analysis was performed using Fisher’s exact test. SHAM: sham-operated group; SS + ISQ: group treated with saline solution and subjected to cardiac ischemia and reperfusion; OME + ISQ: group treated with omeprazole (10 mg/kg) before cardiac ischemia; ISQ + OME: group treated with omeprazole (10 mg/kg) before cardiac reperfusion.

There was no incidence of cardiac arrhythmias (VA and AVB) or LET during the stabilization period in any of the experimental groups, nor in the SHAM group. However, the incidences of VA, AVB, and LET caused by CIR in the SS + CIR group were 85.7%, 78.5%, and 71.4%, respectively. All animals (100%) showed VA and AVB when OME was administered before ischemia (OME + ISQ) and LET (100%) was higher than in the SS + CIR group (*p* = 0.098). On the other hand, the incidences of VA (71.4%), AVB (64.3%) and LET (57.1%) in the ISQ + OME group, where OME was administered after ischemia but before reperfusion, were similar to those in the SS + CIR group, and significantly lower than in the OME + ISQ group (*p* = 0.098; *p* = 0.041; *p* = 0.016, respectively) (Figure 1).

### 2.2. Electrophysiological Ventricular Arrhythmias (VA) and Atrioventricular Block (AVB) and Letahlity Outcomes (LET) in Spontaneously Hypertensive Rats (SHR)

**Figure 2:**
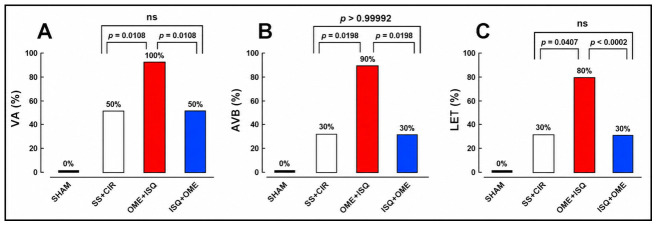
Incidence of (**A**) ventricular arrhythmias (VA), (**B**) atrioventricular block (AVB) and (**C**) lethality (LET) in spontaneously hypertensive rats (SHR); from SHAM, SS + CIR, OME + ISQ and ISQ + OME groups. Results are expressed as percentages obtained with 10–14 animals. Statistical analysis was performed using Fisher’s exact test. SHAM: sham-operated group; SS + ISQ: group treated with saline solution and subjected to cardiac ischemia and reperfusion; OME + ISQ: group treated with omeprazole (10 mg/kg) before cardiac ischemia; ISQ + OME: group treated with omeprazole (10 mg/kg) before cardiac reperfusion. ns= not significant.

To assess the impact of omeprazole on cardiac electrical stability during myocardial ischemia–reperfusion, the incidence of VA, AVB, and LET was quantified across the SHAM, SS + CIR, OME + ISQ, and ISQ + OME groups (Figure 2). No episodes of VA, AVB, or LET were observed during the 15 min stabilization period prior to ischemia in any group. Consistently, the SHAM group remained free of electrophysiological alterations throughout the entire protocol, confirming its suitability as a negative control.

In the SS + CIR animals, ischemia–reperfusion induced substantial electrical instability, with VA in 50%, AVB in 30%, and LET in 30% of animals. In contrast, pre-ischemic omeprazole administration (OME + ISQ) markedly worsened outcomes, with VA in 100%, AVB in 90%, and LET in 80% of animals. Compared with SS + CIR, OME + ISQ showed a significant increase in lethality (*p* = 0.0407, Fisher’s exact test), indicating aggravation when omeprazole was administered in the pre-ischemic period.

When omeprazole was administered immediately before reperfusion (ISQ + OME), the incidences of VA (50%), AVB (30%), and LET (30%) were similar to those in SS + ISQ, with no statistically significant differences between these groups. Importantly, a direct comparison between ISQ + OME and OME + ISQ demonstrated significant reductions in VA (*p* = 0.0108), AVB (*p* = 0.0198), and LET (*p* = 0.0002) when omeprazole was administered during the pre-reperfusion window. These findings indicate that the electrophysiological effects of omeprazole in myocardial ischemia–reperfusion are strongly dependent on the timing of administration relative to the ischemic event.

### 2.3. Biochemical Analysis of Serum Concentrations of Creatine Kinase MB Fraction in Normotensive Wistar Rats (NWR) and Spontaneously Hypertensive Rats (SHR)

**Figure 3:**
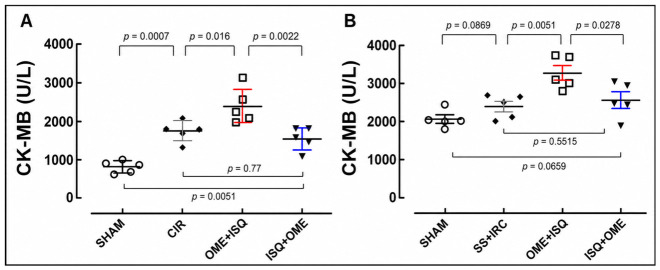
Serum concentrations of creatine kinase-MB (CK-MB) in normotensive Wistar rats (NWR) and spontaneously hypertensive rats (SHR). (**A**) Serum CK-MB concentrations in NWR and (**B**) serum CK-MB concentrations in SHR subjected to cardiac ischemia and reperfusion. Values are expressed as mean ± standard deviation (SD) obtained from five animals per group (*n* = 5). Statistical analyses were performed using one-way ANOVA followed by Tukey’s post hoc test. SHAM: sham-operated group; CIR and SS + IRC: saline-treated animals subjected to cardiac ischemia and reperfusion; OME + ISQ: animals treated with omeprazole (10 mg/kg) before cardiac ischemia; ISQ + OME: animals treated with omeprazole (10 mg/kg) immediately before cardiac reperfusion.

To quantify the extent of myocardial injury induced by omeprazole treatment and cardiac ischemia and reperfusion, serum creatine kinase MB fraction (CK-MB), a well-established biomarker of myocardial necrosis, was measured. Analyses were performed on serum samples obtained from five NWR and five SHR per experimental group (*n* = 5), and the comparative results among SHAM, SS + IRC, OME + ISQ, and ISQ + OME are shown in Figure 3. The MB fraction of the enzyme creatine kinase (CK-MB) is a specific biomarker of myocardial injury widely used in the diagnosis of acute myocardial infarction, which is why its serum concentrations were measured and evaluated in the different experimental groups. For the measurements, 5 samples were collected from each group, as this was the number of samples obtained from the SS + CIR group and, therefore, to ensure an equal number of samples, we decided to evaluate 5 samples from all other experimental groups.

In NWR, cardiac ischemia and reperfusion significantly increased serum CK-MB concentrations in the SS + CIR group (1727 ± 258 U/L) compared with the SHAM animals (808 ± 161 U/L; *p* = 0.0007), confirming the occurrence of myocardial injury. Pretreatment with omeprazole before ischemia (OME + ISQ) further increased serum CK-MB levels (2362 ± 405 U/L) compared with SS + CIR animals (*p* = 0.016), indicating an aggravation of myocardial damage. In contrast, the administration of omeprazole immediately before reperfusion (ISQ + OME) resulted in CK-MB levels (1547 ± 292 U/L) similar to those observed in the SS + CIR group (*p* = 0.77), suggesting no additional increase in myocardial injury (Figure 3A).

In SHR, baseline CK-MB concentrations were higher than those observed in the NWR. Serum CK-MB levels were 2041 ± 231.6 U/L in the SHAM group, 2370 ± 297.0 U/L in the SS + IRC group, 3255 ± 423.8 U/L in the OME + ISQ group, and 2519 ± 290.4 U/L in the ISQ + OME group. No significant differences were observed among the SHAM, SS + IRC, and ISQ + OME groups. However, pretreatment with omeprazole before ischemia (OME + ISQ) promoted a significant increase in serum CK-MB concentrations compared with both SHAM and SS + IRC groups, suggesting enhanced myocardial injury in hypertensive animals exposed to omeprazole prior to ischemia (Figure 3B).

Taken together, these findings indicate that omeprazole administration before ischemia exacerbated myocardial injury in both normotensive and hypertensive animals, whereas administration immediately before reperfusion did not significantly alter CK-MB concentrations compared with the saline-treated ischemia/reperfusion animals (SS + IRC).

### 2.4. Histopathological Analysis of Cardiac Tissue

#### 2.4.1. Histopathological Findings in Normotensive Wistar Rats (NWR)

**Figure 4:**
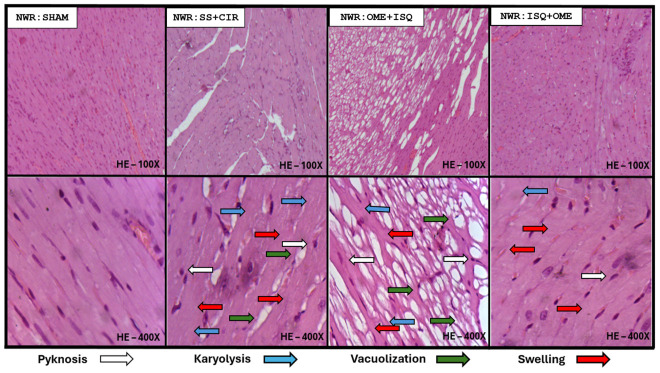
Microscopic photographs of myocardial tissue from normotensive Wistar rats (NWR) from the SHAM, SS + CIR, OME + ISQ, and ISQ + OME groups, stained with hematoxylin and eosin (H&E), at magnifications of 100× (**top** panel) and 400× (**bottom** panel). SHAM group: false-operated; SS + CIR group: rats were treated with 0.9% saline solution before cardiac ischemia and reperfusion; OME + ISQ: rats were treated with OME (10 mg/kg, IV) 1 min before cardiac ischemia and reperfusion; ISQ + OME: rats were treated with OME (10 mg/kg, IV) post-ischemia, 1 min before cardiac reperfusion. Colored arrows indicate the main histopathological alterations observed: Pyknosis (white arrow); Karyolysis (blue arrow); Vacuolization (green arrow); Cellular swelling (red arrow). The control groups (SHAM and SS + CIR) in Figure 4 are from reference [14], used under CC BY 4.0 license (https://www.scielo.br/journal/ramb/about/#about, accessed on 12 June 2026).

No necrosis was observed in the SHAM group, and the cells appeared striated, with well-centered nuclei of normal color and size. In the SS + CIR group, intense coagulation necrosis was observed, with moderate tissue loss, moderate myocytolysis (local loss of myocardial syncytium), moderate vacuolization, intense swelling (increased cell volume due to reversible ionic imbalance and accumulation of water), and moderate pyknosis (decentralized nuclei and chromatin condensation) and/or karyolysis (nuclear loss). In the OME + ISQ group, there was similarly intense coagulation necrosis, with intense tissue loss, with areas of myocytolysis, swollen muscle fibers, intense vacuolization, and cells exhibiting pyknosis or karyolysis. In the ISQ + OME group, on the other hand, no tissue loss was observed, but there was moderate pyknosis, with mild karyolysis (absence of nucleus and cytoplasmic eosinophilia) and swelling, along with areas of reversible lesion (granulation tissue) (Figure 4).

#### 2.4.2. Histopathological Analysis of Cardiac Tissue in SHR

**Figure 5:**
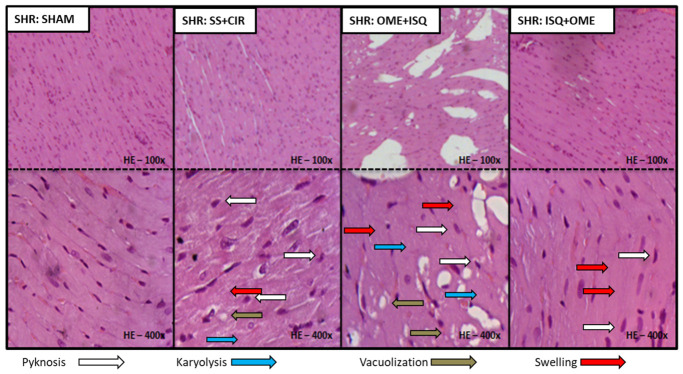
Microscopic photographs of myocardial tissue from Wistar rats (SWR) from the SHAM, SS + CIR, OME + ISQ, and ISQ + OME groups, stained with hematoxylin and eosin (H&E), at magnifications of 100× (**top** panel) and 400× (**bottom** panel). SHAM group: false operated; SS + CIR group: rats were treated with 0.9% saline solution before cardiac ischemia and reperfusion; OME + ISQ: rats were treated with OME (10 mg/kg, IV) 1 min before cardiac ischemia and reperfusion; ISQ + OME: rats were treated with OME (10 mg/kg, IV) post-ischemia, 1 min before cardiac reperfusion. Colored arrows indicate the main histopathological alterations observed: Pyknosis (white arrow); Karyolysis (blue arrow); Vacuolization (brown arrow); Cellular swelling (red arrow).

To characterize myocardial structural alterations induced by ischemia–reperfusion and omeprazole treatment at different time points, histopathological analysis was performed on hematoxylin and eosin (H&E)—stained myocardial sections. Representative findings for the SHAM, SS + CIR, OME + ISQ, and ISQ + OME groups are shown in Figure 5.

Marked morphological differences were observed among the experimental groups. The SHAM group exhibited preserved myocardial architecture, with intact muscle fibers, well-defined cross-striations, centrally located nuclei, and uniform staining, without evidence of necrosis, edema, or structural disorganization, confirming histological integrity in the absence of ischemia.

In the SS + CIR group, cardiac ischemia and reperfusion induced moderate-to-severe coagulative necrosis, characterized by focal tissue loss, myolysis, moderate intracellular vacuolization, pronounced cellular swelling, and nuclear alterations such as pyknosis and karyolysis, indicating significant structural myocardial injury.

Pre-ischemic omeprazole administration (OME + ISQ) further aggravated morphological damage, with extensive necrosis, evident myolysis, marked cellular swelling, intense vacuolization, and frequent nuclei displaying pyknosis and/or karyolysis across multiple myocardial regions. In contrast, the ISQ + OME group exhibited less severe structural impairment. No areas of frank necrosis or overt tissue loss were observed. Instead, mild-to-moderate cellular alterations predominated, including nuclear pyknosis, mild karyolysis, and cytoplasmic swelling, along with areas suggestive of reversible injury and early regenerative features.

Collectively, these findings demonstrate that the extent of myocardial morphological injury is dependent on the timing of omeprazole administration relative to the ischemic event, with greater structural damage observed following pre-ischemic exposure.

### 2.5. Results of Potential Molecular Targets of Omeprazole in Cardiomyocytes According to Molecular Docking Analysis

To provide mechanistic support for the electrophysiological, biochemical, and histopathological findings, molecular docking analyses were performed to evaluate the interactions between omeprazole and cardiovascular targets involved in autonomic regulation, calcium homeostasis, and mitochondrial function. Binding affinity parameters and principal protein–ligand interaction residues are summarized in Table 1.

Overall, omeprazole demonstrated favorable binding affinity to all evaluated targets, with the predicted binding energy score (ΔG) ranging from −6.6 to −8.5 kcal·mol^−1^, consistent with thermodynamically stable interactions. The highest affinities were observed for complexes with 5ZK8 (muscarinic M_2_ receptor) and 6DT0 (mitochondrial calcium uniporter), both exhibiting ΔG = −8.5 kcal·mol^−1^, followed by 4U14 (muscarinic M_3_ receptor; ΔG = −8.4 kcal·mol^−1^).

In the omeprazole–4U14 complex (human muscarinic M_3_ receptor), the ligand established multiple interactions within the binding site, involving aromatic and polar residues such as Tyr A: 529, Tyr A: 506, Tyr A: 148, Trp A: 525, Trp A: 199, Asn A: 513, and Ser A: 151, as well as hydrophobic residues including Leu A: 225 and Val A: 510. This interaction pattern suggests stable accommodation of omeprazole within the receptor binding pocket, supported by hydrophobic contacts and hydrogen bonding.

Similarly, the omeprazole–5ZK8 complex (human muscarinic M_2_ receptor) showed high affinity (ΔG = −8.5 kcal·mol^−1^), involving aromatic, hydrophobic, and polar residues such as tyrosine, tryptophan, phenylalanine, asparagine, and cysteine. The recurrent presence of aromatic residues supports stabilization through π–π and π–hydrophobic interactions.

In the omeprazole–6DT0 complex (mitochondrial calcium uniporter), the binding affinity was equally high (ΔG = −8.5 kcal·mol^−1^), with interactions involving residues such as glutamate, threonine, tyrosine, and tryptophan across distinct protein chains. The multiplicity of polar and charged contacts suggests potential ligand interference within functionally relevant regions associated with mitochondrial calcium influx regulation.

In contrast, the omeprazole–7KL5 complex (calmodulin bound to the RyR_2_ segment) exhibited the lowest affinity among the evaluated targets (ΔG = −6.6 kcal·mol^−1^), indicating comparatively weaker stabilization. Nonetheless, interactions with polar and charged residues such as aspartate, lysine, and asparagine were identified, suggesting possible functional association.

Docking analysis with 8E59 (human L-type calcium channel) revealed intermediate affinity (ΔG = −7.5 kcal·mol^−1^), characterized predominantly by hydrophobic interactions involving valine, isoleucine, methionine, and phenylalanine, along with polar contacts including serine, threonine, and glutamine. This pattern indicates that omeprazole may accommodate within hydrophobic channel regions, potentially influencing conformational dynamics.

Collectively, the molecular docking findings indicate that omeprazole is capable of directly interacting with key proteins involved in autonomic signaling, intracellular calcium regulation, and mitochondrial function. The predicted binding affinities are compatible with biologically plausible interactions, providing a mechanistic support for the extra-gastric cardiovascular effects observed in the experimental CIR model.

**Figure 6:**
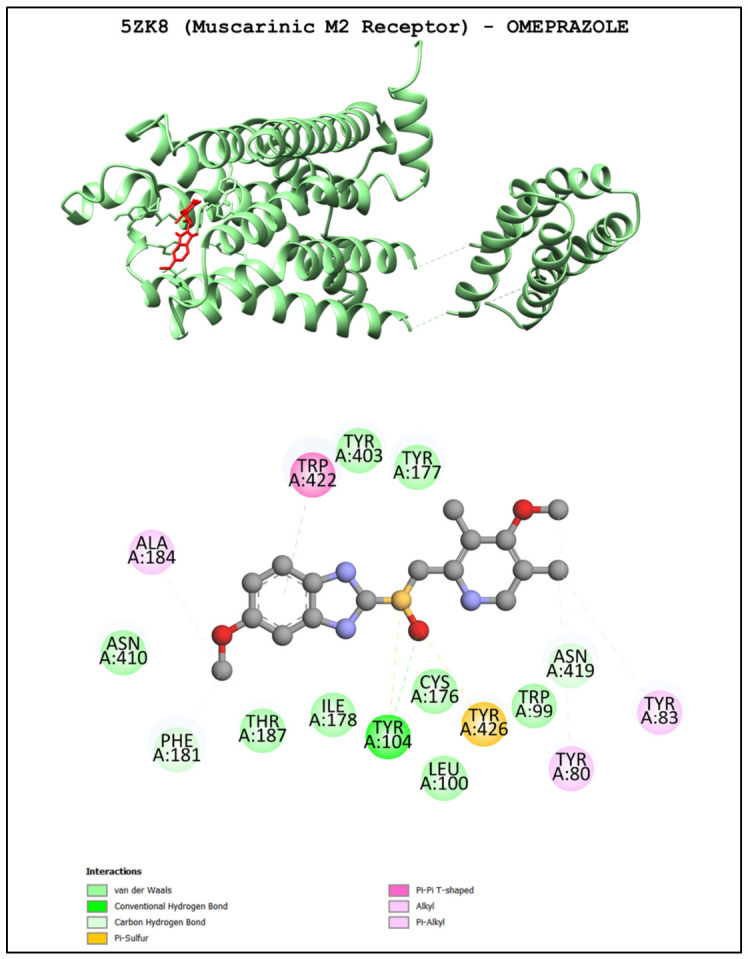
5ZK8 (Muscarinic M2 Receptor)—Omeprazole. Protein structure represented in green and the bound ligand, omeprazole, represented in red. The lower panel shows the two-dimensional interaction map of the ligand-binding site. Colors indicate the type of molecular interaction: van der Waals interactions in light green, conventional hydrogen bonds in green, carbon-hydrogen bonds in pale green, π-sulfur interactions in yellow, π–π T-shaped interactions in magenta, alkyl interactions in purple, and π-alkyl interactions in pink/lavender.

**Figure 7:**
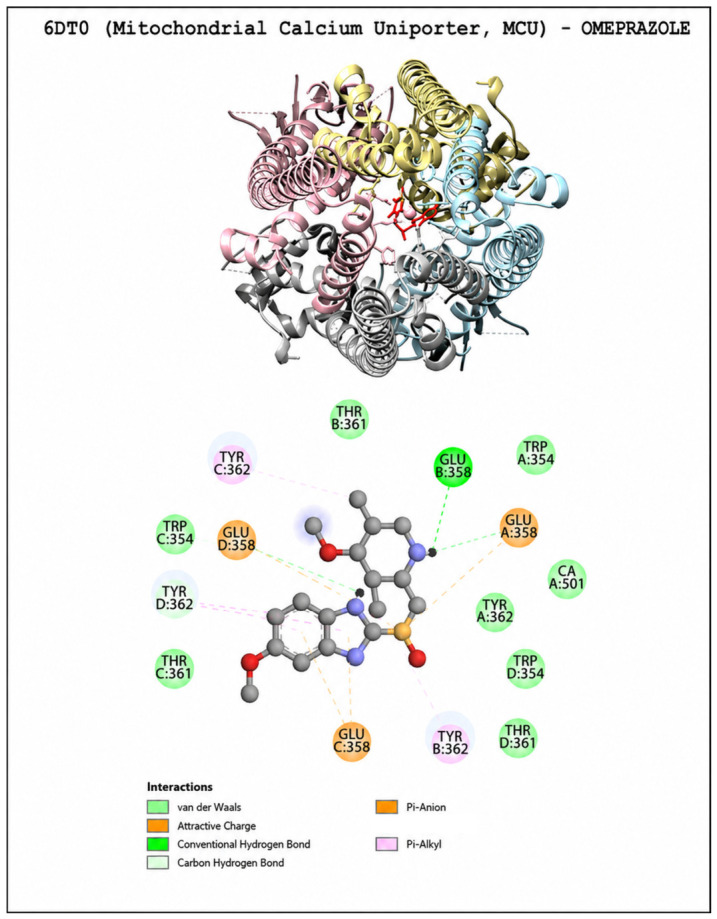
6DT0 (Mitochondrial Calcium Uniporter, MCU)—Omeprazole Complex. The protein is represented by four distinct chains: Chain A (pink), Chain B (khaki), Chain C (blue), and Chain D (gray). The bound ligand, omeprazole, is represented in red in the three-dimensional structure. The lower panel shows the two-dimensional ligand–residue interaction map. The colors indicate different interaction types, including van der Waals, attractive charge, conventional hydrogen bond, carbon–hydrogen bond, π-anion, π–π stacked, and π-alkyl interactions, according to the color-coded legend.

To further illustrate the molecular docking findings, representative three-dimensional models of the omeprazole–target complexes are presented in Figure 6 and Figure 7. The selected structures correspond to the muscarinic M2 receptor (5ZK8) and the mitochondrial calcium uniporter (6DT0), both of which exhibited the highest predicted binding affinities among the evaluated targets (ΔG = −8.5 kcal·mol^−1^). These graphical representations highlight the predicted binding poses of omeprazole within the protein structures and provide a visual depiction of the interactions that may underlie the potential effects of omeprazole on cardiac electrophysiology, calcium homeostasis, and mitochondrial function. Together, these structural models complement the quantitative docking results and provide additional mechanistic insight into the potential cardiovascular actions of omeprazole.

## 3. Discussion

Since their clinical introduction in the late 1980s, proton pump inhibitors (PPIs) have become a cornerstone in the management of acid-related disorders and *Helicobacter pylori* eradication regimens, supported by high efficacy and widespread availability [15,16]. Nonetheless, accumulating evidence has raised concerns regarding the systemic consequences of chronic and often indiscriminate use, including micronutrient deficiencies, infectious complications, renal dysfunction, and potential cardiovascular effects [17,18,19,20,21]. In parallel, observational studies have suggested associations between PPI exposure and increased cardiovascular risk in selected populations [22].

The present study demonstrated that administering OME after ischemia, but before cardiac reperfusion, did not exacerbate CIR-induced functional, biochemical and structural heart damage. However, early administration of OME, prior to CI, resulted in cardiotoxic effects that worsened CIR-induced heart tissue damage and potentially increased lethality (LET). These cardiotoxic effects suggest that the prolonged use of OME may increase the risk of myocardial injury, potentially exacerbating cardiac damage and electrophysiological changes when patients develop acute myocardial infarction (AMI).

Currently, despite technological advances in AMI treatment, many sudden deaths still occur before patients reach hospital emergency care units [4,10]. VAs, specifically ventricular tachycardia, and ventricular fibrillation, as well as the occurrence of either first-, second-, or third-degree AVB, are the primary causes of these unexpected deaths in patients with AMI [4,5,6,7,8,9,10].

Within this framework, the present study demonstrates that the cardiovascular effects of omeprazole in CIR (OME + ISQ) model are critically dependent on the timing of administration relative to the ischemic insult. In our study, we did not observe significant increases in VA and AVB incidence in the OME + ISQ groups, likely due to limited study power, as these rates were already high in untreated SS + CIR animals. Nevertheless, it is noteworthy that 100% of the OME + ISQ NWR showed VA, AVB before succumbing after cardiac reperfusion. Meanwhile, post-ischemia OME administration (ISQ + OME) was considerably safer than OME + ISQ regarding VA, AVB and LET. In spontaneously hypertensive rats (SHR), pre-ischemic administration (OME + ISQ) markedly aggravated electrophysiological instability and mortality, whereas administration immediately prior to reperfusion (ISQ + OME) did not exacerbate injury and significantly improved outcomes when compared with the pre-ischemic regimen.

From an electrophysiological perspective, OME + ISQ was associated with AV in 100% of animals, AVB in 90%, and LET in 80%, compared with 50%, 30%, and 30%, respectively, in SS + ISQ. Direct comparison between OME + ISQ and SS + ISQ revealed significantly increased LET, supporting a deleterious effect when omeprazole precedes coronary occlusion. By contrast, the ISQ + OME exhibited VA, AVB, and LET rates (50%, 30%, and 30%) comparable to those in the SS + ISQ and significantly lower than those in the OME + ISQ.

Subsequent studies further confirmed the presence of myocardial H^+^/K^+^-ATPase in isolated rat cardiomyocytes through a combined methodological approach, including polymerase chain reaction product sequencing, protein characterization by immunocytochemistry and Western blotting, and the functional evaluation of K^+^ transport [23]. Additional evidence has indicated that this proton pump contributes to the positive inotropic, negative chronotropic, and antiarrhythmic effects elicited by three distinct H^+^/K^+^-ATPase inhibitors in isolated rat atria [24]. More recently, the expression of gastric H^+^/K^+^-ATPase was also demonstrated in human and rabbit myocardium by real-time polymerase chain reaction, thereby broadening the current understanding of its tissue distribution and suggesting a potentially relevant role in cardiovascular physiology and pathophysiology [25]. These findings collectively indicate that omeprazole does not exert uniform cardiotoxicity but rather displays context- and timing-dependent effects.

In isolated rat hearts, administering 200 µg of OME before cardiac ischemia exhibited a cardioprotective effect on CIR-induced lesions. This effect was attributed to the regulation of transmembrane H^+^/K^+^ gradients, which are modified during CIR and contribute to repolarization changes, as reflected in the amplitude and duration of the T wave [26,27]. Fluorometric analysis and magnetic resonance imaging of isolated guinea pig hearts validated the occurrence of relevant and abrupt intracellular ionic changes in cardiomyocytes [27,28,29]. These ionic changes, mainly in Na^+^ and Ca^2+^, regulate several cell processes directly or indirectly related to cardiac arrhythmias and the mortality of animals subjected to the CIR protocol [14,28].

Both in NWR and SHR biochemical and histopathological analyses corroborated this interpretation. CK-MB levels were significantly elevated in the OME + ISQ compared with the SHAM and ISQ + OME, indicating greater myocardial necrosis. Histologically, OME + ISQ demonstrated extensive necrosis, pronounced myolysis, intense cellular swelling, and widespread nuclear pyknosis and karyolysis. In contrast, the ISQ + OME exhibited only mild-to-moderate structural alterations without frank necrosis, suggesting partial preservation of myocardial integrity. The convergence of functional, biochemical, and morphological findings strengthens the internal consistency of the model.

Of note, these results align with previous publications from our group. In normotensive Wistar rats, pre-ischemic pantoprazole administration produced maximal incidences of VA, AVB, and LET (100%/100%/100%), respectively [14]. Similarly, prior investigations demonstrated cardiotoxic effects of omeprazole in CIR models and emphasized the relevance of temporal pharmacological modulation [30]. Taken together, these data support the hypothesis of a class-related effect of PPIs when administered before ischemia.

Furthermore, from a mechanistic standpoint, the DDAH–ADMA–NOS–NO axis provides a coherent explanatory framework. Nitric oxide (NO) plays a pivotal role in coronary vasodilation, mitochondrial respiration, and electrophysiological stability [31,32]. Asymmetric dimethylarginine (ADMA), an endogenous NOS inhibitor, is strongly associated with endothelial dysfunction and increased cardiovascular risk [6,33]. Experimental studies have demonstrated that PPIs inhibit DDAH activity, thereby elevating ADMA levels and reducing NO bioavailability [6,34,35]. Furthermore, clinical investigations have reported impaired endothelial function and alterations in NO-related metabolites among PPI users [34,35].

Reduced NO bioavailability may promote NOS uncoupling, enhance reactive oxygen species (ROS) generation, and exacerbate mitochondrial dysfunction and intracellular Ca^2+^ overload [36,37]. This pro-oxidative milieu facilitates electrical instability and arrhythmogenesis, offering a mechanistic explanation for the marked increase in VA, AVB, LET, and CK-MB observed in OME + ISQ. In addition, NO directly modulates ion channels and Ca^2+^-handling proteins, including L-type Ca^2+^ channels and RyR_2_ thereby influencing excitation–contraction coupling and arrhythmic susceptibility [38,39,40].

Conversely, the pathophysiological landscape during reperfusion differs substantially, being characterized by abrupt ROS generation and Ca^2+^ overload. Under these conditions, omeprazole may exert antioxidant and cytoprotective effects. Experimental studies have demonstrated the anti-inflammatory and antioxidant properties of PPIs in myocardial injury models [41]. Moreover, omeprazole has been shown to activate aldehyde dehydrogenase 1A1 (ALDH1A1), enhancing the detoxification of reactive lipid aldehydes and mitigating oxidative stress-induced cellular damage [42]. These mechanisms are particularly relevant during reperfusion, when oxidative injury peaks, and may account for the comparatively favorable phenotype observed in ISQ + OME.

Complementing the in vivo findings, molecular docking analysis revealed a multi-target pharmacological profile for omeprazole. The drug exhibited favorable binding affinities with muscarinic receptors M_2_ (5ZK8; ΔG = −8.5 kcal·mol^−1^) and M_3_ (4U14; ΔG = −8.4 kcal·mol^−1^), the mitochondrial calcium uniporter (6DT0; ΔG = −8.5 kcal·mol^−1^), the L-type Ca^2+^ channel (8E59; ΔG = −7.5 kcal·mol^−1^), and the calmodulin–RyR_2_ complex (7KL5; ΔG = −6.6 kcal·mol^−1^). Docking procedures followed validated computational methodologies described in the literature [43,44,45].

Interaction with the mitochondrial calcium uniporter (MCU) is particularly noteworthy. Mitochondrial Ca^2+^ overload is a critical determinant of permeability transition pore opening and cardiomyocyte death during reperfusion. Our group previously demonstrated that pharmacological MCU blockade confers significant cardioprotection in ischemia–reperfusion models [13,46,47]. Accordingly, the omeprazole–MCU interaction may contribute to the attenuation of reperfusion injury in ISQ + OME.

Similarly, the affinity for L-type Ca^2+^ channels suggests the potential modulation of Ca^2+^ influx, a recognized cardioprotective strategy that limits intracellular overload [47,48,49]. Predicted interactions with muscarinic receptors may further influence autonomic balance and atrioventricular conduction [50,51]. Even the moderate predicted interaction with the calmodulin–RyR_2_ complex may carry biological relevance, given the high regulatory sensitivity of Ca^2+^ release channels [49].

In summary, the integration of in vivo and in silico data supports a multifactorial model in which omeprazole modulates NO signaling, redox homeostasis, Ca^2+^ handling, mitochondrial function, and autonomic pathways. The net cardiovascular effect appears deleterious when the drug is administered prior to ischemia but neutral or partially protective when delivered immediately before reperfusion, underscoring the principle of context-dependent pharmacology.

## 4. Materials and Methods

### 4.1. Animals

Male normotensive Wistar rats (NWR) and spontaneously hypertensive rats (SHR; Rattus norvegicus albinus), 12- to 16-week-old (290–320 g), were housed under a controlled temperature (21 ± 2 °C) and a 12 h light/dark cycle, with ad libitum access to standard chow and water [14,49]. All procedures were approved by the Ethics Committee on Animal Use of Escola Paulista de Medicina/Universidade Federal de São Paulo (protocol numbers: 9728120921, approved on 14 October 2021 and 1439030226, approved on 16 April 2026) and conducted in accordance with institutional guidelines.

### 4.2. Normotensive Wistar Rats (NWR) Were Randomly Allocated into Four Groups

- SHAM (*n* = 10): The NWR were submitted to identical surgical manipulation without left anterior descending (LAD) coronary occlusion.
- SS + CIR (*n* = 14): The NWR were treated with a 0.9% saline solution intravenously 1 min before cardiac ischemia and reperfusion (CIR).
- OME + ISQ (*n* = 14): The NWR were treated with omeprazole, 10 mg/kg, intravenously 1 min before cardiac ischemia and reperfusion (CIR).
- ISQ + OME (*n* = 14): The NWR were treated with omeprazole, 10 mg/kg, intravenously, immediately before cardiac reperfusion and ten minutes after the onset of cardiac ischemia.

### 4.3. Spontaneously Hypertensive Rats (SHR) Were Randomly Allocated into Four Groups

- SHAM (*n* = 10): The SHR were submitted to identical surgical manipulation without left anterior descending (LAD) coronary occlusion.
- SS + CIR (*n* = 14): The SHR were treated with a 0.9% saline solution intravenously 1 min before cardiac ischemia and reperfusion (CIR).
- OME + ISQ (*n* = 14): The SHR were treated with omeprazole, 10 mg/kg, intravenously 1 min before cardiac ischemia and reperfusion (CIR).
- ISQ + OME (*n* = 14): The SHR were treated with omeprazole, 10 mg/kg, intravenously, immediately before cardiac reperfusion and ten minutes after the onset of cardiac ischemia.

### 4.4. Cardiac Ischemia and Reperfusion (CIR) Model

Cardiac ischemia and reperfusion (CIR) was induced as previously described [45,46]. Rats were anesthetized intraperitoneally with ketamine (75 mg/kg) and xylazine (8 mg/kg), intubated with a 14G catheter (Jelco), and mechanically ventilated (EFF 312 ventilator, Insight Equipamentos Científicos, Ribeirão Preto, SP, Brazil) using parameters adjusted to body weight.

A left thoracotomy was performed at the fourth or fifth intercostal space. The LAD coronary artery was identified and occluded using a nylon suture placed around the vessel and tightened against a small polyethylene tube, producing complete interruption of blood flow for 10 min (cardiac ischemia). The ligature was then released to allow 75 min of cardiac reperfusion. At the end of the protocol, the animals were euthanized by exsanguination via the abdominal aorta, and cardiac tissue was collected for histopathological analysis.

### 4.5. Electrocardiographic Monitoring and Arrhythmia Analysis

ECG monitoring was performed as previously described [29,46]. After a 15 min stabilization period (baseline), subcutaneous needle electrodes were placed in the limbs and connected to a biopotential amplifier for continuous ECG recording throughout the ischemia and reperfusion. Effective LAD occlusion was confirmed by typical ECG changes, including ST-segment elevation and increased R-wave amplitude [29,45]. Body temperature was maintained at 37.5 °C using a heated surgical table and infrared lamps, and monitored by a rectal thermometer [29,45].

Signals were acquired using AqDados 7.02 and analyzed using AqDAnalysis 7 (Lynx Tecnologia Ltd.a., São Paulo, SP, Brazil) [14,29,45,46]. The following outcomes were assessed: heart rate (beats/min), the incidence of ventricular arrhythmias (VA), atrioventricular block (AVB), and lethality (LET). Ventricular arrhythmias were defined as episodes of ventricular fibrillation, torsades de pointes, or ventricular tachycardia according to established ECG criteria.

### 4.6. Biochemical Assessment of Myocardial Injury

Blood samples were collected from the abdominal aorta of five animals per group (*n* = 5) immediately after the ISQ/RC protocol. Samples (3–4 mL) were collected in siliconized tubes, centrifuged at 2500 rpm for 40 min at 5 °C, and the serum was stored at −20 °C until analysis. Serum creatine kinase MB-fraction (CK-MB) activity was measured using a kinetic UV enzymatic kit (Vida Biotecnologia, Belo Horizonte, MG, Brazil) with a spectrophotometric reading at 340 nm, following the manufacturer’s instructions.

### 4.7. Histopathological Analysis

Hearts were fixed in 10% buffered formalin, processed for paraffin embedding, and sectioned (4–5 μm). Sections were stained with hematoxylin and eosin (H&E) and examined under a light microscope (Zeiss Axio Imager A2^®^, Carl Zeiss Microscopy GmbH, Jena, Germany) at 400× and 1000× magnification. The histopathological assessment was performed by an experienced pathologist blinded to group allocation and included a descriptive comparison of typical injury features: vascular congestion/hyperemia, nuclear pyknosis, an interstitial inflammatory infiltrate, cardiomyocyte degeneration/vacuolization, a loss of cross-striations, and interstitial edema.

### 4.8. Molecular Docking Analysis

The three-dimensional (3D) structure of omeprazole was built using GaussView 5 and optimized using Gaussian 09W.

Geometry optimization was performed using density functional theory (DFT) with the B3LYP functional and the 6-311G basis set [42,43,44]. Only geometry optimization calculations were carried out, and vibrational frequency calculations were not performed [42,43,44].

Protein 3D structures were obtained from the Protein Data Bank with the following identifiers: 4U14 (x = 5.272; y = 24.294; z = 344.486), 5ZK8 (x = 187.885; y = 24.340; z = 525.974), 6DT0 (x = −147.156; y = 147.194; z = 201.835), 7KL5 (x = −12.956; y = 21.789; z = 15.218), and 8E59 (x = 151.444; y = 172.657; z = 149.554). Docking simulations were performed using AutoDock 4.2, and protein–ligand preparation was conducted with AutoDock Vina. The protein structures retrieved from PDB were subjected to a preprocessing procedure, which included the removal of co-crystallized ligands, water molecules, and non-essential ions. Partial Gasteiger charges were assigned after the addition of polar and nonpolar hydrogens to both the protein and the ligand. A cubic grid box (30 × 30 × 30 Å) was centered on the protein binding site to define the docking search space. The default docking parameters were used, with the number of modes set to 100 and the exhaustiveness parameter set to 50. The resulting poses were clustered according to their RMSD, and the final complexes were selected based on the best binding energies combined with visual inspection [42,43,44].

### 4.9. Statistical Analysis

VA, AVB, and LET were expressed as percentages and compared using Fisher’s exact test. CK-MB values were expressed as mean ± SD and compared using one-way ANOVA followed by Tukey’s multiple comparisons test. Analyses were performed in GraphPad Prism 8.0 (GraphPad Software, San Diego, CA, USA), adopting *p* < 0.05 as the significance threshold.

## 5. Conclusions

In the experimental model of myocardial ischemia and reperfusion employed in this study, the cardiovascular effects of omeprazole were shown to be strongly dependent on the timing of administration and the underlying pathophysiological context. Pre-ischemic administration was associated with marked electrophysiological instability, increased lethality, and the exacerbation of myocardial injury. In contrast, administration immediately before reperfusion reduced the incidence of arrhythmias, decreased lethality, and attenuated biochemical and histopathological alterations, suggesting a cardioprotective effect restricted to the reperfusion phase.

## Figures and Tables

**Table 1:** Predicted binding affinities (ΔG, kcal·mol^−1^) and main protein–ligand interactions between omeprazole and cardiovascular targets.

(Ligand–Protein Complex)	ΔG_liga_^um^ (kcal·mol^−1^)	Mode	Ligand Interactions with Protein Residues
Omeprazole-Muscarinic M3 receptor (4U14)	−8.4	1	Tyr A: 529, Tyr A: 506, Trp A: 525, Asn A: 513, Thr A: 231, Val A: 510, Tyr A: 148, Leu A: 225, Thr A: 234, Trp A: 199, Ala A: 235, Ala A: 238, Ser A: 151
Omeprazol-Muscarinic M2 receptor (5ZK8)	−8.5	1	Tyr A: 177, Tyr A: 403, Trp A: 422, Ala A: 184, Asn A: 410, Phe A: 181, Thr A: 187, Ile A: 178, Tyr A: 104, Cys A: 176, Leu A: 100, Tyr A: 426, Trp A: 99, Tyr A: 80, Asn A: 419, Tyr A: 83
Omeprazol-Mitochondrial calcium uniporter (6DT0)	−8.5	1	Thr B: 361, Glu B: 358, Trp A: 354, Glu A: 358, Ca A: 501, Tyr A: 362, Trp D: 354, Thr D: 361, Tyr B: 362, Glu C: 358, Thr C: 361, Tyr D: 362, Trp C: 354, Glu D: 358, Tyr C: 362
Omeprazol-Calmodulin-RyR2 complex (7KL5)	−6.6	1	Asp A: 65, Pro A: 67, Phe A: 17, Lys A: 14, Phe A: 66, Ser A: 18, Gly A: 24, Asp A: 21, Gly A: 26, Thr A: 27, Asn A: 61
Omeprazol-L-type calcium channel (8E59)	−7.5	1	Val A: 1148, Ile A: 1456, Ala A: 1452, Phe A: 1453, Phe A: 1147, Met A: 1449, Phe A: 1095, Ser A: 1098, Tyr A: 1135, Gln A: 1026, Val A: 1139, Thr A: 1023, Thr A: 1022, Met A: 1019, Met A: 1144

The letters A, B, C, and D shown after the amino acid residue indicate the respective protein chain.

## Data Availability

The original contributions presented in this study are included in the article. Further inquiries can be directed to the corresponding authors.
